# Mast cells selectively produce inflammatory mediators and impact the early response to *Chlamydia* reproductive tract infection

**DOI:** 10.3389/fimmu.2023.1166068

**Published:** 2023-04-17

**Authors:** Animamalar Mayavannan, Emily Shantz, Ian D. Haidl, Jun Wang, Jean S. Marshall

**Affiliations:** ^1^ Department of Microbiology and Immunology, Dalhousie University, Halifax, NS, Canada; ^2^ Canadian Center for Vaccinology, Izaak Walton Killam (IWK) Health Centre, Halifax, NS, Canada; ^3^ Department of Pathology, Dalhousie University, Halifax, NS, Canada

**Keywords:** mast cells, *Chlamydia trachomatis*, *chlamydia* reproductive tract infection, Toll-like receptor 2, inflammatory mediators, mucosal immunity

## Abstract

**Introduction:**

*Chlamydia trachomatis* (*C. trachomatis*) is a Gram-negative obligate intracellular bacterium that causes reproductive tract complications in women, including ectopic pregnancies and tubal factor infertility. We hypothesized that mast cells, which are common at mucosal barriers, may contribute to responses to *Chlamydia* infection and aimed to define human mast cell responses to *C. trachomatis*.

**Methods:**

Human cord blood-derived mast cells (CBMCs) were exposed to *C. trachomatis* to assess bacterial uptake, mast cell degranulation, gene expression, and production of inflammatory mediators. The role of formyl peptide receptors and Toll-like receptor 2 (TLR2) were investigated using pharmacological inhibitors and soluble TLR2. Mast cell-deficient mice and littermate controls were used to examine the *in vivo* role of mast cells in influencing the immune response to *Chlamydia* infection in the female reproductive tract.

**Results:**

*C. trachomatis* bacteria were taken up by human mast cells but did not replicate efficiently inside CBMCs. *C. trachomatis*-activated mast cells did not degranulate but maintained viability and exhibited cellular activation with homotypic aggregation and upregulation of ICAM-1. However, they significantly enhanced the gene expression of *IL1B*, *CCL3*, *NFKB1*, *CXCL8*, and *IL6*. Inflammatory mediators were produced, including TNF, IL-1β, IL-1RA, IL-6, GM-CSF, IL-23, CCL3, CCL5, and CXCL8. Endocytic blockade resulted in reduced gene expression of *IL6*, *IL1B*, and *CCL3*, suggesting *C. trachomatis* induced mast cell activation in both extracellular and intracellular locations. The IL-6 response to *C. trachomatis* was reduced when CBMCs were treated with *C. trachomatis* coated with soluble TLR2. Mast cells derived from TLR2-deficient mice also demonstrated a reduced IL-6 response to *C. muridarum*. Five days following *C. muridarum* infection, mast cell-deficient mice showed attenuated CXCL2 production and significantly reduced numbers of neutrophils, eosinophils, and B cells in the reproductive tract when compared with mast cell-containing littermates.

**Discussion:**

Taken together, these data demonstrate that mast cells are reactive to *Chlamydia* spp. through multiple mechanisms that include TLR2-dependent pathways. Mast cells also play an important role in shaping *in vivo* immune responses in *Chlamydia* reproductive tract infection through both effector cell recruitment and modification of the chemokine microenvironment.

## 1 Introduction

*Chlamydia trachomatis* (*C. trachomatis*) is an obligate intracellular bacterium that causes reproductive tract, respiratory, and ocular infections. *Chlamydia* disproportionately affects women, with a global incidence rate of 4.2% in women and 2.7% in men (1), and presents as an asymptomatic infection in up to 70% of affected women (2). Without diagnosis and treatment, it ascends to the upper reproductive tract and may cause serious complications such as chronic pelvic pain, pelvic inflammatory disease, and hydrosalpinx, resulting in tubal factor infertility or ectopic pregnancies (3, 4). The nature of the cellular and mediator responses that lead to damaging inflammation and the mechanisms of resolution of post-infection uterine tract pathologies are not well understood. However, regulating such responses could be critical for the development of effective prevention or treatment for *C. trachomatis*-induced pathology.

The human uterus is an immune-privileged mucosal site that is constantly responding to a variety of triggers, such as hormones including estrogen and progesterone, microbiota, and foreign antigens (5). Mast cells are granulated, sentinel, bone marrow-derived immune cells. Mast cell progenitors are derived from hematopoietic stem cells, which very quickly pass through the circulatory system and become tissue residents with a differentiated phenotype dependent on the tissue microenvironment (6). Mast cells are deeply embedded in the cervix, fallopian tubes, and endometrium and myometrium linings of the human uterus, thus becoming key players of mucosal immune responses in the reproductive tract. Mast cell subtypes populating the uterus have been characterized (7–10), but their responses to uterine infections have been vastly understudied.

Apart from their key role in allergic responses, mast cells have pivotal roles in host defense against a wide variety of bacterial, parasitic, and viral infections (11–14). They recognize pathogenic stimuli through a variety of surface and intracellular receptors, including pattern recognition receptors, Fc receptors, and complement receptors. They respond to antigens by releasing cytokines, chemokines, lipid mediators, histamines, and proteases either by degranulation or *de novo* synthesis (15–17). Mast cell products have impacts on the immune sequelae and clearance of infection, including the activation and mobilization of antigen-presenting cells, phagocytic activity of macrophages and neutrophils, activation of the endothelium, vascular permeability, impacts on epithelial barrier function, and the induction of humoral immune responses (18–21). Mast cells are implicated in the recruitment of immune effector cells in response to multiple types of infection and injury through the production of chemokines and cytokines (22, 23). In experimental models of bacterial infection, mast cells are critical for the initial recruitment of immune effector cells and may have direct anti-bacterial impacts such as phagocytosis and subsequent destruction of bacteria (24). However, the longer-term impacts of mast cell responses to agents that cause chronic infection are less well understood and may include the promotion of chronic inflammatory processes. Currently, mast cell responses to *Chlamydia* have only been investigated in a murine model of respiratory *C. pneumoniae* infection and have been described to aid leukocyte recruitment and accommodate elevated bacterial growth (25).

For complex organisms such as *Chlamydia* sp., several molecular pathways may be engaged in response to a bacterial challenge, providing a challenge for elucidating mechanisms of mast cell–pathogen interaction. Both Toll-like receptors (TLRs) and formyl peptide receptors (FPRs) have been described as having leading roles in mast cell responses to bacteria (26, 27). Human and mouse mast cells express a range of TLRs, which, depending on the tissue location and source of cells, often include TLR1, TLR2, TLR4, TLR6, and TLR9 with roles in antibacterial host defense (28–30). TLR2, dimerized with either TLR1 or TLR6, is widely expressed by murine and human mast cells at multiple locations and mediates multiple potentially protective responses (28, 31–33).

In the current study, the response of human and mouse mast cells to *Chlamydia* infection was examined, and the impact of inhibiting the FPR and TLR2 pathways on key mast cell mediator responses was evaluated. We also investigated the importance of mast cells to inflammatory events in response to an *in vivo* murine *Chlamydia muridarum* infection in mast cell-deficient mice. Our findings demonstrate a complex response that includes limited mast cell uptake of *Chlamydia* bacteria but no evidence of ongoing productive infection, and a diverse cytokine, chemokine, and adhesion molecule response in the absence of classical degranulation or leukotriene C4 generation. *In vivo* results reveal a critical role for mast cells in chemokine production and immune cell recruitment into the reproductive tract at early stages following *Chlamydia* reproductive tract infection.

## 2 Methods

### 2.1 Mice

C57BL/6J (strain No. 000664) and TLR2^−/−^ C.129(B6)-Tlr2*^tm1Kir^*/J (strain No. 022507) mice were obtained from Jackson Laboratories (Bar Harbor, Maine, USA) and bred in the house within the Carleton Animal Care Facility (Dalhousie University). Cpa3-Cre; Mcl-1^fl/fl^ mice were obtained from Dr. S. Galli and M. Tsai (Stanford University) and bred on-site. For *Chlamydia muridarum in vivo* infection experiments, 6–8-week-old female mice and age-matched littermate controls were used. All procedures were approved by the Dalhousie University Committee on Laboratory Animals according to Canadian Council for Animal Care guidelines. Mice were pretreated with subcutaneous injection of 2.55 mg medroxyprogesterone acetate injectable suspension DEPO-PROVERA (Pfizer, New York, NY, USA, DIN 00585092) at days 10 and 3 to synchronize their estrous cycle and subsequently inoculated intravaginally with 300,000 inclusion forming units (IFU) of monolayer-derived *Chlamydia muridarum* at day 0. Animals were euthanized at different time points, and female reproductive tract tissue, iliac lymph nodes (ILN), and spleen tissues were harvested for immunological and histological analyses.

### 2.2 Processing of animal tissues

Female reproductive tract tissues were either fixed in 4% formalin for histological analysis, snap-frozen for later RNA extraction, or digested in collagenase A and collagenase D (Roche, Millipore Sigma, Oakville, ON, Canada) for 1 h at 37°C with constant agitation. Digested tissues were then pressed through a 40-μm cell strainer. Spleen and lymph node tissues were pressed directly on a 70-μm cell strainer, and red blood cell lysis was carried out on spleen tissues with an ammonium chloride lysis solution. Single-cell suspensions were washed in RPMI 5% fetal bovine serum (FBS), pelleted at 300×*g* for 10 min at 4°C, and used for downstream applications such as flow cytometry. For protein analysis, animal tissues were weighed and homogenized using Qiagen Tissue Ruptor II in PBS and added to a protease inhibitor cocktail (Sigma-Aldrich, St. Louis, MO, USA).

### 2.3 *Chlamydia* propagation, purification, and quantification

*Chlamydia* was propagated and purified using mouse fibroblast McCoy cells (McCoy B, CRL-1696™) as described previously (34, 35). Briefly, supernatant-derived *Chlamydia* was purified by ultracentrifugation of supernatants harvested from the infected McCoy cells at 22,500×*g* for 1 h at 4°C. Monolayer-derived *Chlamydia* was purified following the lysis of McCoy monolayers by sonication and density gradient separation with 30% of the Isovue solution (ISOVUE^®^ iopamidol injection) and 50% sucrose dissolved in 30 mM Tris HCl at 22,500×*g* for 1 h at 4°C. Purified *Chlamydia* was quantified by an IFU assay. McCoy cells infected with serially diluted standards or samples were fixed in 100% methanol and stained with Gimenez solution (1 g of carbol fuchsin dissolved in 10 ml of ethanol, 4% phenol dissolved in distilled water) at room temperature for 20 s. The cells were then counterstained with 0.8% Malachite Green solution, and IFU was assessed visually. *Chlamydia trachomatis* serovar E (ATCC VR 348B™) was used for human mast cell experiments and *Chlamydia muridarum* (ATCC VR-123™) was used for murine mast cell experiments and *in vivo* infection experiments.

### 2.4 Primary human cord blood-derived mast cell culture

Pure cultures of human primary mast cells were derived from mononuclear cells isolated from a human umbilical cord. Human umbilical cord blood samples were obtained from consenting mothers, following approval from the Research Ethics Board (REB #1005110) of the IWK Health Centre in Halifax, Canada. Buffy coat with mononuclear leukocytes was harvested after centrifugation over Lymphoprep, and cells washed with PBS. Red blood cells were lysed, and the remaining cells were cultured for 3 weeks in StemSpan™ SFEM serum-free medium for the expansion of hematopoietic cells (StemCell Technologies, Vancouver, BC, Canada) supplemented with 100 ng/ml recombinant human stem cell factor (SCF) (Peprotech) and 10 ng/ml IL-6 (BioLegend, San Diego, CA, USA). During the first week of culture, 10 ng/ml recombinant human IL-3 (BioLegend, San Diego, CA, USA) was added. After 3 weeks, cells were switched to “CBMC medium,” consisting of RPMI medium with 10% FBS, 100 ng/ml of SCF, 10 ng/ml of IL-6, and 50 µM of 2-mercaptoethanol. After 5 weeks of culture, cord blood-derived mast cells (CBMC) were stained for CD117 (anti-human CD117 (c-Kit), Invitrogen, Waltham, MA, USA) and analyzed by flow cytometry for mast cell purity. Mast cells were used for experiments when the purity was determined to be >95%. CBMCs were rested in CBMC medium with 10 ng/ml of hSCF (in place of 100 µg/ml of hSCF) for 16–24 h prior to *in vitro* experiments.

### 2.5 Murine bone marrow-derived mast cells

Pure cultures of mouse primary mast cells were derived from mononuclear cells isolated from mouse bone marrow. Femur and tibia bones were harvested from mice, and bone marrow was flushed using RPMI 10% FBS. The cell suspension was then passed through a 40-μm strainer and centrifuged at 300×*g* at 4°C for 10 min. Cells were cultured in a “murine bone marrow-derived mast cell (BMMC) medium” consisting of 10% FBS, 1% penicillin–streptomycin, 50 μM of 2-mercaptoethanol, 200 nM of prostaglandin E2 (PGE_2_) (Sigma-Aldrich, St.Louis, MO, USA), and 15% of WEHI 3B cell culture supernatant as a source of IL-3. At 5 weeks, murine BMMC purity was assessed by flow cytometry staining for CD117 (anti-mouse CD117 (c-Kit), Invitrogen, Waltham, MA, USA). BMMCs were used for experiments when mast cell purity was determined to be >95%. BMMCs were rested in BMMC medium without PGE_2_ for 16–24 h prior to *in vitro* experiments.

### 2.6 Transmission electron microscopy

CBMCs were washed and plated on a 24-well plate in RPMI supplemented with 10% FBS, 20 mM of HEPES, 10 ng/ml of hSCF, and 100 µg/ml of soybean trypsin inhibitor (STI) at a cell density of 4 × 10^6^ cells per well. McCoy cells at a cell density of 2 × 10^6^ per well were used, in parallel, as positive controls for *C. trachomatis* infection. Mast cells and McCoy cells were treated with *C. trachomatis* MOI = 0.5 or MOI = 1 or MOI = 20 and incubated at 37°C in a 5% CO_2_. At time points 6 and 40 h post-infection, cells were harvested and resuspended in 1 mL of 2.5% glutaraldehyde in 0.1 M of sodium cacodylate and stored at 4°C. Cells were visualized on a 120-kV JEOL 1239 Transmission Electron Microscope at the CORES facility at Dalhousie University.

### 2.7 β-hexosaminidase assay

CBMCs were washed in HEPES-Tyrode’s buffer and plated at a density of 2×10^6^ cells/ml and treated with *C. trachomatis* MOI = 0.5, 1, or 5 for 15 min or 1 h at 37°C in a 5% CO_2_ incubator. Culture medium alone was used as a negative control, and calcium ionophore A23187 (Sigma-Aldrich, St.Louis, MO, USA) was used as a positive control at a concentration of 10^−6^ M. Cell pellets and supernatants were harvested at the respective time points, and mast cell degranulation was assessed by quantifying β-hexosaminidase released as described in Schwartz et al. (36). Percent degranulation was calculated using the following method:

% of degranulation = (OD of supernatant − OD of control) ÷ [(OD of supernatant − OD of control) + (OD of pellet − OD of control] × 100.

### 2.8 Mast cell treatments and infections

CBMCs were set up at a density of 1 × 10^6^ cells/ml in CBMC infection medium, which contained RPMI medium, 2.5% FBS, 15 mM of HEPES, 10 ng/ml of hSCF, and 100 µg/ml of STI for *Chlamydia* infection experiments. BMMCs were set up at a density of 1 × 10^6^ cells/ml in BMMC infection medium, containing 2.5% FBS, 15 mM of HEPES, 100 µg/ml of STI, and 3 ng/ml of recombinant IL-3 (BioLegend, San Diego, CA, USA). CBMCs and BMMCs were treated with *C. trachomatis* or *Chlamydia muridarum* (*C. muridarum*), respectively, at MOI = 0.5 or MOI = 1 or MOI = 5 or with medium alone (control) for 24 to 48 h. For electron microscopy experiments, a higher dose of *C. trachomatis* MOI = 20 was used.

To block the activation of FPRs, CBMCs were preincubated with FPR1 antagonist BOC-MLF (Tocris Bioscience, Bio-Techne, Toronto, ON, Canada) or FPR2 antagonist PBP 10 (Tocris Bioscience, Bio-Techne, Toronto, ON, Canada) at 1 or 10 µM concentrations for 30 min at 37°C in a 5% CO_2_ incubator before being infected with *C. trachomatis*. To block the activation of TLR2, *C. trachomatis* was incubated with 0.2 or 1 µg of sTLR2 (Sino Biologicals, Wayne, PA, USA) for 30 min at 37°C and then added to the CBMCs. Controls were set up in parallel with CBMCs treated with vehicle controls at the highest concentration used for treatments. Endocytosis inhibitor cytochalasin D (Sigma-Aldrich, St. Louis, MO, USA) was used at concentrations of 1.25, 2.5, and 5 µg/ml.

### 2.9 RNA extractions

Mast cells were harvested in TRIzol™ reagent (Invitrogen, Waltham, MA, USA), and RNA extractions were carried out using RNeasy Plus kit (Qiagen, Germantown, MD, USA) according to the manufacturer’s instructions.

### 2.10 RT-PCR, quantitative PCR, and PCR array

In each reverse transcriptase reaction, 150–300 ng of purified RNA was used to generate cDNA. Following genomic DNA elimination with gDNA wipe-out buffer (Qiagen, Germantown, MD, USA) at 42°C for 2 min, cDNA was prepared by setting up an RT-PCR reaction with RNA template, Quantiscript reverse transcriptase, Quantiscript RT buffer, and RT primer mix (Qiagen, Germantown, MD, USA) according to the manufacturer’s instructions.

Quantitative PCR (qPCR) reactions were set up with 1× SSo Advanced™ Universal SYBR^®^ Green Supermix (Bio-Rad, Mississauga, ON, Canada), 250–500 nM of forward and reverse primers (Qiagen, Germantown, MD, USA or Bio-Rad, Mississauga, ON, Canada), and nuclease-free water in Bio-Rad CFX96 touch real-time PCR detection system. qPCR data were analyzed using CFX Maestro™ software. Gene expression values were determined using the formula ΔCq = Cq of the gene of interest − Mean of Cq of housekeeping genes, and normalized expression was then calculated as 2^−(ΔCq)^. A predesigned 384-well PCR panel (Bio-Rad, acute inflammation response H384) was used to screen uninfected *vs*. *C. trachomatis*-infected mast cell gene responses according to the manufacturer’s instructions.

### 2.11 Luminex array

Human and mouse premixed multiplex magnetic Luminex^®^ assay kits were purchased from R&D Systems (R&D Systems, Minneapolis, MN, USA, Cat. No. LXSAHM-24 and No. LXSAMSM-12) and run according to the manufacturer’s instructions using provided reagents. Plates were read on a Bio-Rad Bio-Plex 200 system.

### 2.12 Cytokine ELISAs

Cytokine and chemokine concentrations were determined using ELISA kits (eBioscience, Thermo Fisher Scientific, Waltham, MS, USA, R&D, and Peprotech, Cranbury, NJ, USA) according to the manufacturer’s instructions.

### 2.13 Flow cytometry

Single-cell suspensions from *in vitro* cultures or processed animal tissues were stained with a fixable viability dye for 30 min, followed by blocking in 50 µl of 5% FBS in PBS for 20 min at 4°C. Cells were washed and pelleted by centrifugation at 300×g for 10 min at 4°C and stained with 100 µl of antibody cocktail mix at 4°C for 30 min in FACS buffer containing 2% FBS and 20 mM of sodium azide (Sigma-Aldrich) in PBS. The cells were then washed twice in FACS buffer, fixed with 1% paraformaldehyde at pH 7.2–7.4, and resuspended in FACS buffer before being acquired in a BD LSR Fortessa cytometer or BD FACSymphony cytometer. Data were analyzed using FlowJo™ v10.8.1 software. The FACS antibodies and staining reagents used are described in **Supplementary Table S1**. Appropriate isotype controls and fluorescent minus one (FMO) controls were used to define the expression of certain markers.

### 2.14 Toluidine blue staining

Formalin-fixed paraffin-embedded tissues were deparaffinized and stained with 0.5% toluidine blue in 0.1 N HCl solution for 48 h, rinsed with 0.033 N HCl and distilled water, mounted with DPX, and visualized under a brightfield microscope.

### 2.15 Statistical analysis

All statistical analyses were performed using the GraphPad Prism v9.4.1 software. All data are presented as mean ± SEM. *p*-values <0.05 were considered statistically significant. The specific statistical approach used for each experiment was dependent on data distribution and design, as noted in the figure legends.

### 2.16 Interactome analysis

Protein–protein interaction networks were generated on string-db.org and interpreted as described in von Mering et al. (37) and Szklarczyk et al. (38).

## 3 Results

### 3.1 *Chlamydia trachomatis* is taken up but does not effectively replicate within human mast cells

**Figure 1:**
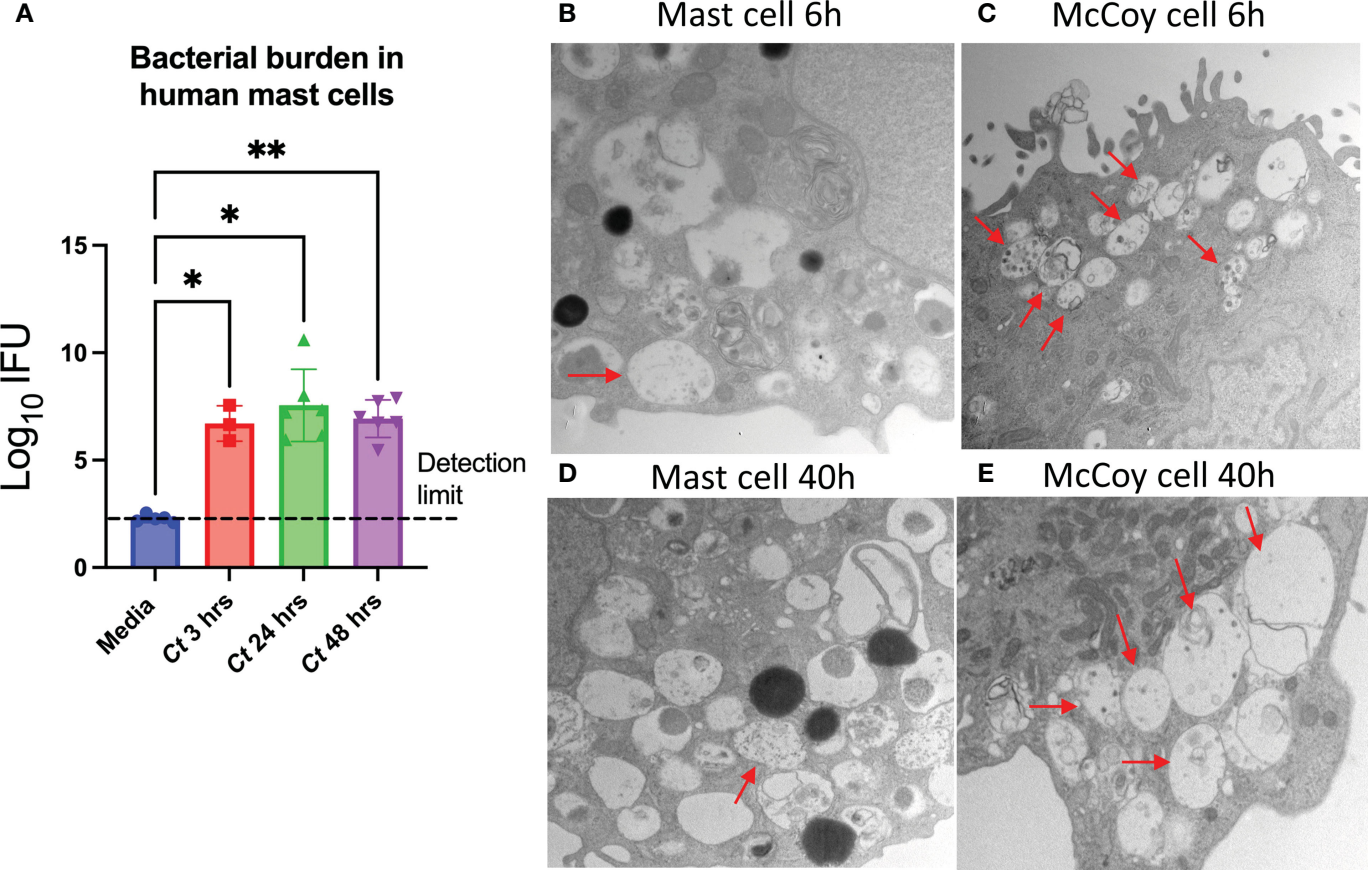
*C trachomatis* is taken up by human mast cells but does not effectively replicate. **(A)** CBMCs were treated with *C trachomatis* MOI = 1 and paired medium controls. Total genomic DNA was isolated from the cell pellets at 3, 24, and 48 h post-treatment, and bacterial content was measured by qPCR amplification of the chlamydial 16S gene. The data represent two independent experiments with *n* = 3 to 6 donors. ^*^ *p* < 0.05 and ^**^ *p* < 0.01, as determined by Sidak’s multiple comparisons tests. **(B–E)** CBMCs were incubated with *C trachomatis* (MOI = 20), and McCoy cells were incubated with *C trachomatis* (MOI = 0.5). At 6 h **(B, C)** and 40 h **(D**, **E)**, *Chlamydia* inclusion bodies were visualized by transmission electron microscopy. Images are representative of duplicate samples prepared for each condition.

*Chlamydia* requires a permissive host cell to replicate and expand. In addition to epithelial cells, immune cells such as dendritic cells, monocytes, macrophages, and neutrophils have been reported to be permissive to *Chlamydia* infection; however, human mast cells have not been previously examined (39). To assess if mast cells could host *Chlamydia*, CBMCs were exposed to *C. trachomatis* MOI = 1, and the cell pellets were harvested at 3, 24, and 48 h post-treatment. qPCR to detect the *C. trachomatis* 16S gene in DNA extracted from the cell pellets of *C. trachomatis*-treated cells showed a significant increase in bacterial genomic content in *C. trachomatis*-treated cells compared to paired diluent-treated controls. However, there was no difference in the levels of bacterial DNA detected between 3 and 48 h post-treatment, indicating that *C. trachomatis* was taken up by the mast cells but the cells did not accommodate *C. trachomatis* replication (**Figure 1A**).

*Chlamydia* has a biphasic life cycle that includes both infectious elementary bodies (EBs) and metabolically active reticulate bodies (RBs). Following endocytosis of EBs, EBs differentiate into RBs within membrane-bound organelles called inclusion bodies, then replicate, mature, and exit through exocytosis as mature EBs (40). Transmission electron microscopy was used to examine if *C. trachomatis* remained bound to the surface of the mast cells as EBs or if the infectious form was endocytosed. CBMCs were treated with a high dose of *C. trachomatis* MOI = 20 for 6 or 40 h, and McCoy cells were infected in parallel with a lower dose (*C. trachomatis* MOI = 0.5) as a positive control. Electron-dense EBs measuring 0.2–0.4 µm in diameter were detected in <10% of CBMCs compared to >50% of McCoy cells (**Figures 1B, C**). Furthermore, McCoy cells presented with multiple early inclusions per cell, whereas this was rarely seen in CBMCs.

At 40 h post-infection of a typical *C. trachomatis* life cycle, the presence of membrane-bound mature inclusion bodies—which in some cases contained both EBs and larger, less electron-dense RBs—was evaluated. Inclusion bodies were detected in a subset of CBMCs, although only <5% of cells had these characteristics (**Figures 1D, E**). Overall, these observations suggest that *C. trachomatis* can be taken up by mast cells. However, the low incidence of inclusion bodies despite treatment with a high dose of *C. trachomatis* is consistent with mast cells being permissive to infection but resistant to *C. trachomatis* replication.

### 3.2 Human mast cells do not undergo degranulation but exhibited homotypic aggregation and upregulation of ICAM-1

**Figure 2:**
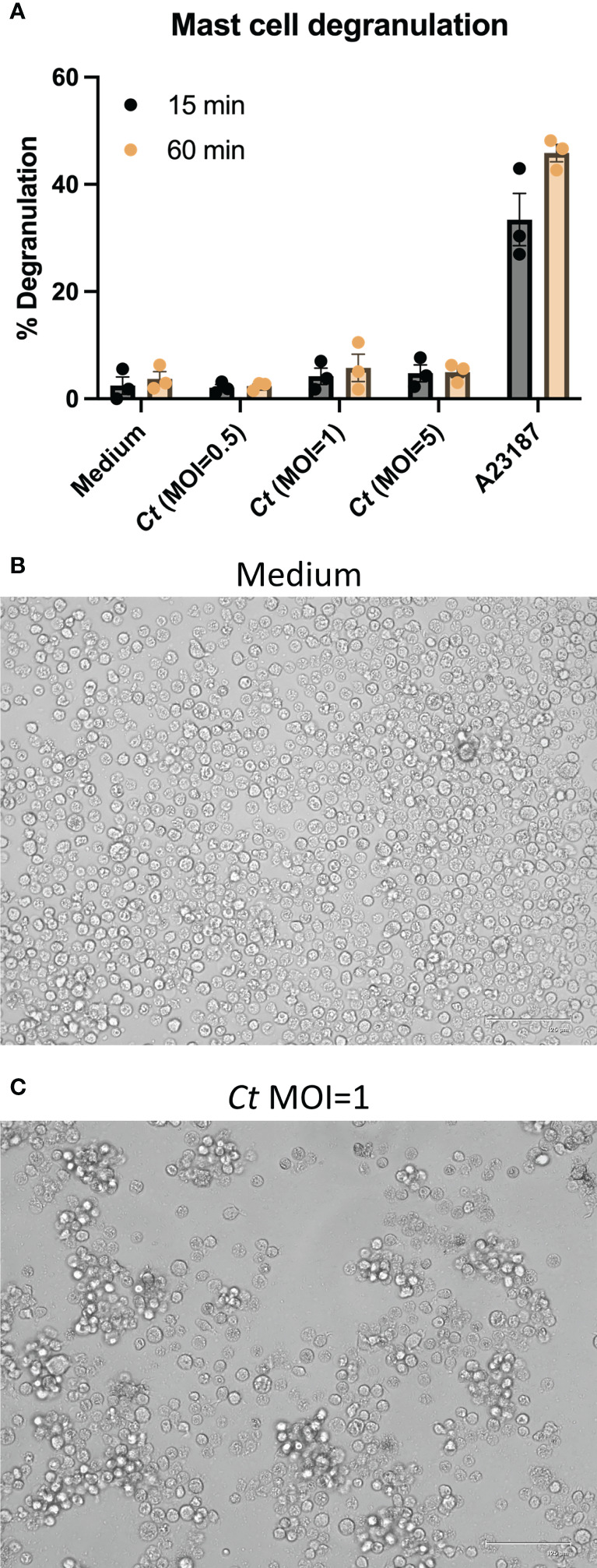
Human mast cells do not undergo degranulation but exhibit cellular activation with homotypic aggregation. **(A)** CBMCs were incubated with *C trachomatis* MOI = 0.5, 1, and 5, A23187 (calcium ionophore), or medium alone for 15 or 60 min. β-hexosaminidase released into culture supernatants was measured relative to β-hexosaminidase in cell pellets. Data are presented as the mean % degranulation ± SEM. The data represent two independent experiments with *n* = 3 donors **(B**, **C)**. CBMCs were treated with *C trachomatis* MOI = 1 for 24 h and images of *C trachomatis*-treated cells **(C)** and paired medium controls **(B)** were acquired under an inverted phase-contrast microscope. Images are representative of two independent experiments with *n* = 3 donors.

Mast cells can degranulate and secrete proteases, histamine, and cytokines and produce lipid mediators in response to various stimuli. To determine whether human mast cells undergo degranulation in response to *C. trachomatis*, CBMCs were infected with varying doses of *C. trachomatis* (MOI = 0.5, 1, and 5), and the release of the granule product β-hexosaminidase was assessed. At 15 and 60 min post-stimulation, no increase in degranulation was observed in *C. trachomatis-*treated mast cells (**Figure 2A**). This indicates that human mast cells do not degranulate, in the short term following *C. trachomatis* infection.

CBMCs treated with *C. trachomatis* at MOI = 1 exhibited homotypic aggregation (**Figures 2B, C**), which could be readily observed as cell clumping. The regulation of adhesion molecules following *C. trachomatis* infection was examined by flow cytometry following staining with antibodies against CD54 (ICAM-1), CD49b (integrin alpha-2), and CD49c (integrin alpha-3). While the expression levels of CD49b and CD49c (**Supplementary Figures S1B, C**) remained unchanged, ICAM-1 was upregulated in *C. trachomatis*-treated mast cells (**Supplementary Figure S1A**). The expression pattern of LFA-1 on mast cells was subsequently examined. CBMCs had a low baseline expression of LFA-1, which was not modified substantially by *C. trachomatis* treatment (**Supplementary Figure S1D**).

### 3.3 Human mast cells have enhanced expression of selected effector genes following *C. trachomatis* exposure

To further assess mast cell responses to *C. trachomatis*, the regulation of 89 acute inflammatory genes (listed under **Supplementary Table S2**) was screened using a PCR array. RNA isolated from two independent CBMC donors treated with *C. trachomatis* was paired with diluent-treated control cell RNA from the same donors. Genes with a twofold change in expression following infection were classified as upregulated genes. Genes with a fold change ranging from 1.5 to 2 were classified as moderately increased. Genes with a fold change below 0.5 were classified as downregulated genes.

Numerous effector genes were upregulated in *C. trachomatis*-activated mast cells. *IL6*, *IL1B*, *CXCL8*, *TNF*, *CXCL2*, *PTGES*, and *CCL2* were upregulated in both donors (**Supplementary Figure S2**). *IL6ST*, *IL1A*, *IL1RN*, *SERPINA1*, *S100A9*, *TNFRSF1A*, *IL13*, *CXCL10*, *CCR6*, *F3*, *CCR2*, *CX3CR1*, and *ICAM1* were upregulated in one of the two donors screened. *TNFSF4*, *FPR2*, *CXCR3*, *IL10, TLR2*, and *NFKB1* were moderately increased with a fold change ranging from 1.5 to 2 in both donors (**Supplementary Figure S2**), and *ICAM1*, *CALCA*, *S100A9*, *CRP*, *TFRC*, *SAA1*, *SERPINA1*, *INS*, *HBA1*, *IL1RN*, and *CEBPB* were moderately increased in at least one of the donors. *CD14*, *C5AR1*, *SIGIRR*, *ITGB2*, *IL18*, *IL6R*, *GATA3*, *CCR1*, *A2M*, and *TLR4 were* downregulated in both donors (**Supplementary Figure S2**), and *TF*, *CXCR4*, *HP*, *CEBPB*, *TFRC*, *FCGR1A*, *AHSG*, *TNFRSF1A*, *F2R*, *C3AR1*, *AKT1*, *MAPK14*, *TGFB1*, *ITGAM*, *F2RL1*, *HBA2*, *APOE*, *ELANE*, and *MPO* were downregulated in one of the two donors assessed.

These results indicate a robust interaction between *C. trachomatis* and mast cells, resulting in the activation of several intracellular inflammatory processes and pathways, and underscore a high donor-to-donor variability in the immune responses elicited.

**Figure 3:**
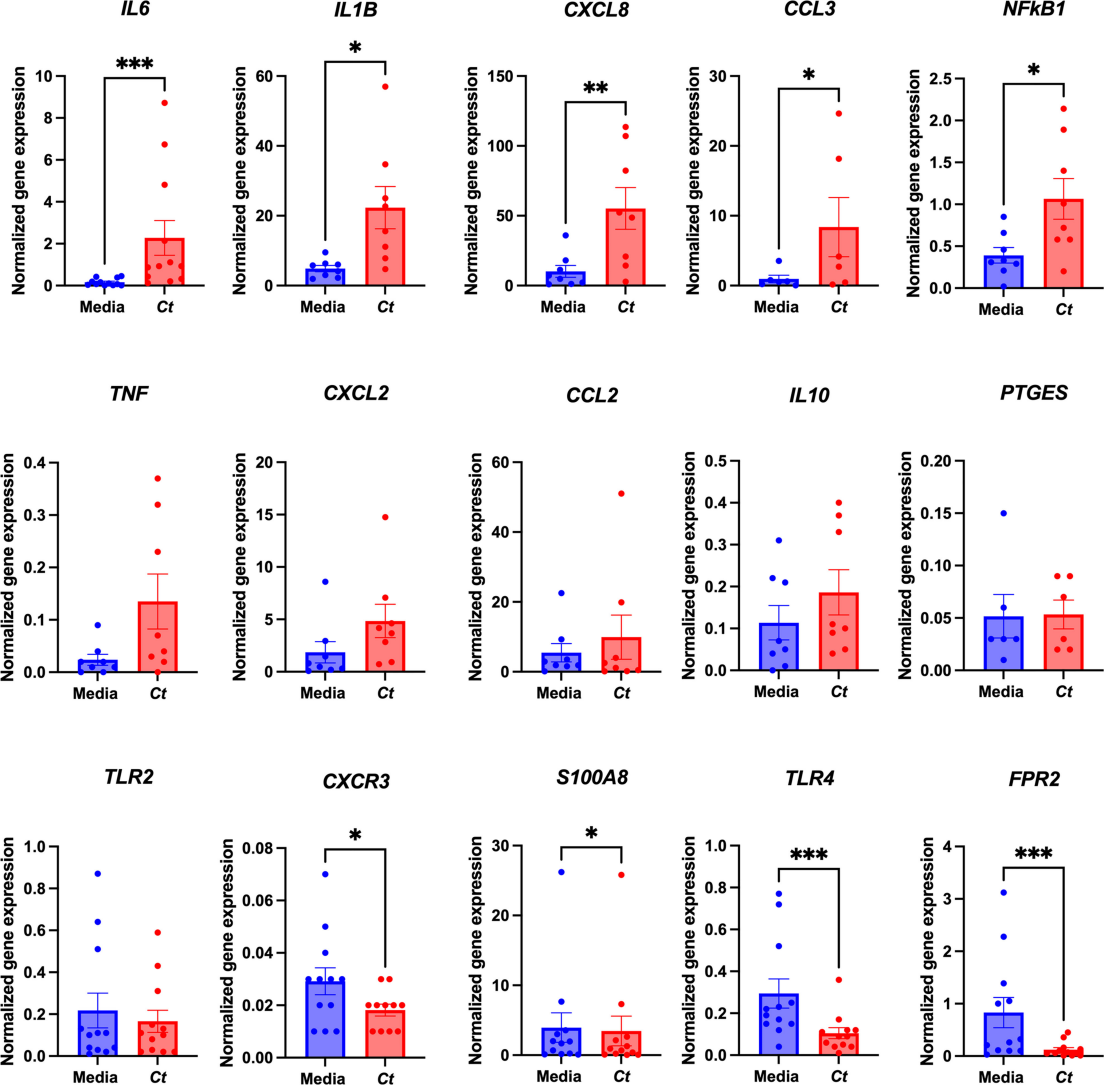
*C. trachomatis*-treated mast cells upregulate the expression of various effector genes. Gene expression analysis of 89 acute inflammatory genes of CBMCs treated with *C. trachomatis* MOI = 1 and paired medium controls was carried out using a PCR array. qPCRs for selected genes from the array were carried out on *C. trachomatis*-treated CBMCs from multiple donors and paired medium controls. Gene expression was normalized to two reference genes, *GUSB* and *HPRT*, and data are presented as 2^−△Cq^. The data are graphed as mean ± SEM. Graphs represent three individual experiments, and each point represents an individual donor. ^*^ *p* < 0.05, ^**^ *p* < 0.01, and ^***^ *p* < 0.001, as determined by a nonparametric Wilcoxon signed-rank test.

Additional targeted qPCR studies on *C. trachomatis*-treated mast cells and paired diluent-treated controls from several donors were completed to verify the impact of *C. trachomatis* treatment on selected genes from the array analysis. *IL6* (*p* = 0.0005, *n* = 12), *IL1B* (*p* = 0.0156, *n* = 8), *CXCL8* (*p* = 0.0078, *n* = 8), *CCL3* (*p* = 0.0312, *n* = 6), and *NFKB1* (*p* = 0.0156, *n* = 8) were significantly upregulated. CXCR3 and S100A8 were significantly downregulated. There were no statistically significant changes in the expression levels of *TNF*, *CXCL2*, *CCL2*, *IL-10*, and *PTGES* (**Figure 3**). While the gene for the pattern recognition receptor TLR2 was stably expressed, genes for TLR4 and FPR2 were downregulated following *C. trachomatis* treatment.

### 3.4 Human mast cells produced cytokines and chemokines following exposure to *C. trachomatis*

**Figure 4:**
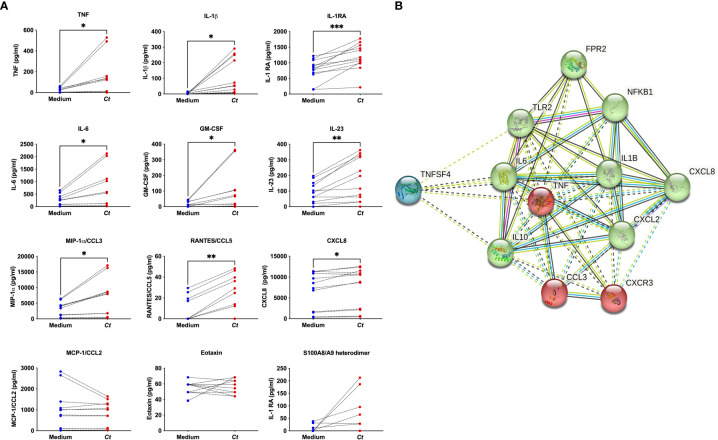
*C. trachomatis*-treated mast cells produced several mediators. Supernatants from CBMCs treated with *C trachomatis* MOI = 1 or paired medium controls were analyzed for the presence of 29 cytokines and chemokines by a multiplex assay. S100A8/A9 heterodimer protein was measured by ELISA **(A)**. Data represent *n* = 6 to 10 donors from three independent experiments. Data are graphed as the mean ± SEM. ^*^ *p* < 0.05, ^**^ *p* < 0.01, and ^***^ *p* < 0.001, as determined by a nonparametric Wilcoxon signed-rank test. An interactome analysis was generated on the STRING database of selected mediators following *C trachomatis* treatment and shows three clusters grouped by green, red, and blue nodes with an average local clustering coefficient = 0.893 **(B)** Edges between the nodes represent protein-to-protein associations indicating known interactions (from curated databases or experimentally determined) or predicted interactions (from gene neighborhood, gene fusions, and co-occurrence) or other predictions through text mining, co-expression, and protein homology.

To evaluate cytokine and chemokine mediator production, supernatants from *C. trachomatis*-activated mast cell cultures at 24 h post-infection were analyzed for 29 protein mediators by a multiplex assay. *C. trachomatis*-treated mast cells produced TNF, IL-1β, IL-6, GM-CSF, IL-23, CCL3, CCL5, CXCL8, and IL-1RA (**Figure 4A**). In addition to the cytokine and chemokine mediators, we also assessed the production of S100A8/A9, a calcium-binding protein called calprotectin, a key player in inflammation and a suppressor of mast cell degranulation (41). While the expression of S100A8 had been observed to be downregulated at a gene level, the S100A8/A9 heterodimer, a stable form of the protein, was upregulated in six out of eight donors screened with a *p*-value of 0.0625 (**Figure 4A**).

A STRING interactome analysis of selected *C. trachomatis*-induced genes, cytokines, and chemokines was performed to assist with data interpretation and inform consideration of key immunological pathways and potential mechanisms of mast cell- *C. trachomatis* interaction (42). The generated interactome showed three clusters (**Figure 4B**), with pattern recognition receptors *FPR2* and *TLR2*, inflammatory pathway molecule *NFKB1*, and inflammatory mediators *IL6*, *IL1B*, *IL10*, *CXCL8*, and *CXCL2* clustered together. We, therefore, hypothesized that mast cell–*C. trachomatis* interaction occurred through pattern recognition receptors and the production of inflammatory mediators downstream of the NF-κB pathway.

### 3.5 Mast cell–*C. trachomatis* interaction occurs at both the surface and intracellular levels and by mechanisms that include Toll-like receptor 2-dependent pathways

**Figure 5:**
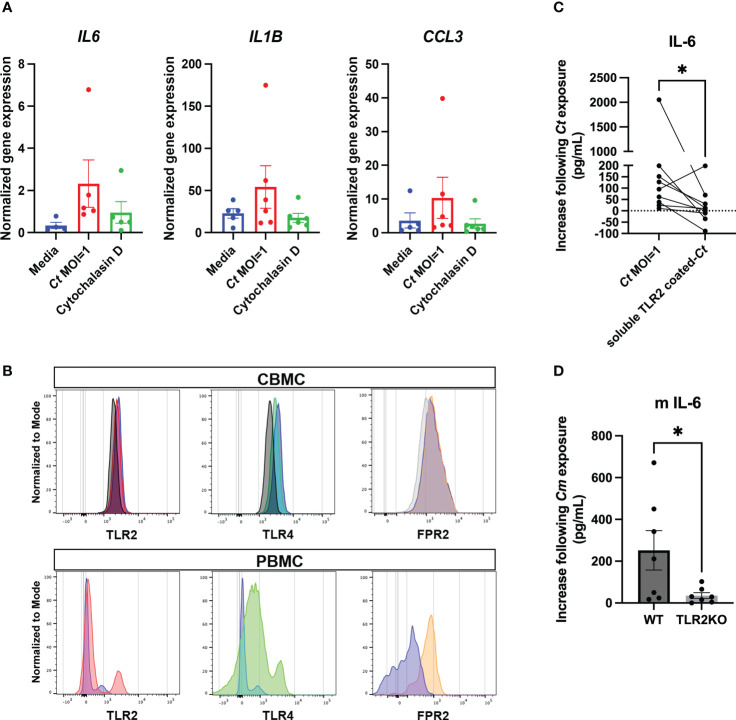
Mast cell *C. trachomatis* interaction occurs at both the surface and intracellular levels by mechanisms that include TLR2-dependent pathways. CBMCs treated with the endocytosis inhibitor cytochalasin D (1.25 µg/ml) prior to *C. trachomatis* exposure were analyzed for *IL6*, *IL1B*, and *CCL3* gene expression **(A)**. Data represents *n* = 6 donors from two independent experiments and are graphed as mean ± SEM. Surface and/or intracellular expression of pattern recognition receptors TLR2, TLR4, and FPR2 was determined by flow cytometry **(B)**. CD117+ CBMCs were gated after the selection of single and live cells, and TLR2, TLR4, and FPR2 expression on *C. trachomatis-*treated versus control mast cells were analyzed by histogram overlays on isotype controls or FMO controls. Shown are example histograms of TLR2, TLR4, and FPR2 expression in *C. trachomatis*-treated CBMCs, shown in red, green, and orange colors, respectively. Medium-only controls are shown in violet color, and isotype or FMO controls are shown in black or grey color histogram overlays. Similarly, PBMCs were gated following the selection of leukocytes, single cells, and live cells, and TLR2, TLR4, and FPR2 expression on PBMCs was analyzed by histogram overlays on isotype controls or FMO controls. TLR2, TLR4, and FPR2 expression on PBMCs are shown in red, green, and orange color overlays, respectively, and isotype or FMO controls are shown in violet overlays. IL-6 in supernatants from CBMCs treated with either sTLR2-coated *C. trachomatis* or pure *C. trachomatis* was measured by ELISA **(C)**. Each dot represents an individual donor and data are graphed as mean ± SEM. Statistical significance was determined by a nonparametric Wilcoxon signed-rank test. IL-6 response in murine mast cells from TLR2-deficient mice and TLR2^+/+^ controls following exposure to *Chlamydia muridarum* (*C. muridarum*) at MOI = 1 was measured by an ELISA **(D)**. Each dot represents an individual experiment, and the data show pooled readouts from seven independent experiments with supernatants harvested either at 24 or 48 h post-*C. muridarum* infection. Statistical significance was determined by an unpaired *t*-test. (^*^ *p* < 0.05).

Given the differing distributions of pattern recognition receptors between the cell surface and endocytic compartments, the nature of the interaction of *C. trachomatis* with mast cells was further investigated. The uptake of bacteria was blocked with the endocytosis inhibitor cytochalasin D. Endocytic blockade resulted in a reduction in gene expression of *IL6*, *IL1B*, and *CCL3* (**Figure 5A**). These results suggest that the bacterial–mast cell interaction occurs at both the cell surface and intracellularly following endocytosis, consistent with interactions with known pattern recognition receptors.

The expression of TLR2, TLR4, and FPR2 on CBMC was characterized by flow cytometry (**Figure 5B**) in *C. trachomatis*-treated mast cells and controls. PBMCs were used as a positive control to screen PRR expression. A high degree of donor-to-donor variability was observed in the expression patterns of these molecules. TLR2 and FPR2 were further functionally investigated.

The ectodomain portion of TLR2, termed soluble TLR2, has been described to modulate immune responses at human mucosal interfaces (43). Preincubating *C. trachomatis* with sTLR2 selectively blocked the production of IL-6 (**Figure 5C**) but did not alter gene expression or the production of other soluble mediators examined (data not shown). sTLR2 also effectively blocked TLR2-dependent *IL6* gene expression in mast cells stimulated by Pam_3_CSK_4_ (**Supplementary Figure S3**). The defined antagonists BOC-MLF and PBP-10 were used to block FPR1 and FPR2, respectively. Inhibition of FPRs did not alter the production of IL-6, GM-CSF, CXCL8, or CCL3, suggesting that the *C. trachomatis–*mast cell interaction responsible for enhanced production of these mediators occurred independently of FPR activation (**Supplementary Figure S4**). Genetic single-nucleotide polymorphisms in the TLR2 gene could potentially explain the high degree of variability observed in the human dataset. To overcome this, we further investigated the TLR2 dependency in the IL-6 response to *Chlamydia* using murine mast cells.

Murine mast cells derived from TLR2-deficient mice also showed attenuated IL-6 production compared to the TLR2-sufficient controls (**Figure 5D**), confirming that IL-6 production as a result of *Chlamydia*–mast cell interaction occurs partially *via* a TLR2-dependent pathway. Furthermore, the IL-6 response in control murine mast cells underscores the robust responsiveness of mast cells to *Chlamydia* spp. and warrants further investigations in an *in vivo* reproductive tract infection.

### 3.6 Mast cells modulate the chemokine milieu of the female reproductive tract

The role of mast cells has only been previously studied in a lung infection with *Chlamydia pneumoniae* in a *Kit* gene-deficient mouse model (25). Mast cell responses in the reproductive tract during *Chlamydia* infection have not been previously studied. While the *Kit*-deficient Wsh mice lack mast cells, there are also effects on other myeloid cell populations. We investigated the role of mast cells in a reproductive tract *Chlamydia* infection model using Cpa3-Cre; Mcl-1^fl/fl^ ‘Hello Kitty’ (HK) mice (44). These mice are severely deficient in mast cells, while there are no significant changes observed in other immune cell types. Methods have not been developed to selectively reconstitute mast cells in the female reproductive tract, limiting our analyses to mice with or without mast cells since birth. Given the gene response and soluble mediator production by human mast cells in response to *Chlamydia*, we hypothesized that mast cell functions could modulate and orchestrate immune responses in the female reproductive tract. HK mice and littermate controls were pretreated with progesterone hormone to synchronize their estrous cycle, and we looked at the immune responses at baseline and at the acute time points of days 3 and 5 following intravaginal infection with *C. muridarum*. Female reproductive tract homogenates from HK mice and littermate controls were screened on a multiplex assay for soluble mediators, including chemokines that would recruit monocytes, macrophages, neutrophils, T and B cells, effector cytokines, and inflammatory mediators, including IFN-γ, IL-12p70, TNF, and ICAM-1.

**Figure 6:**
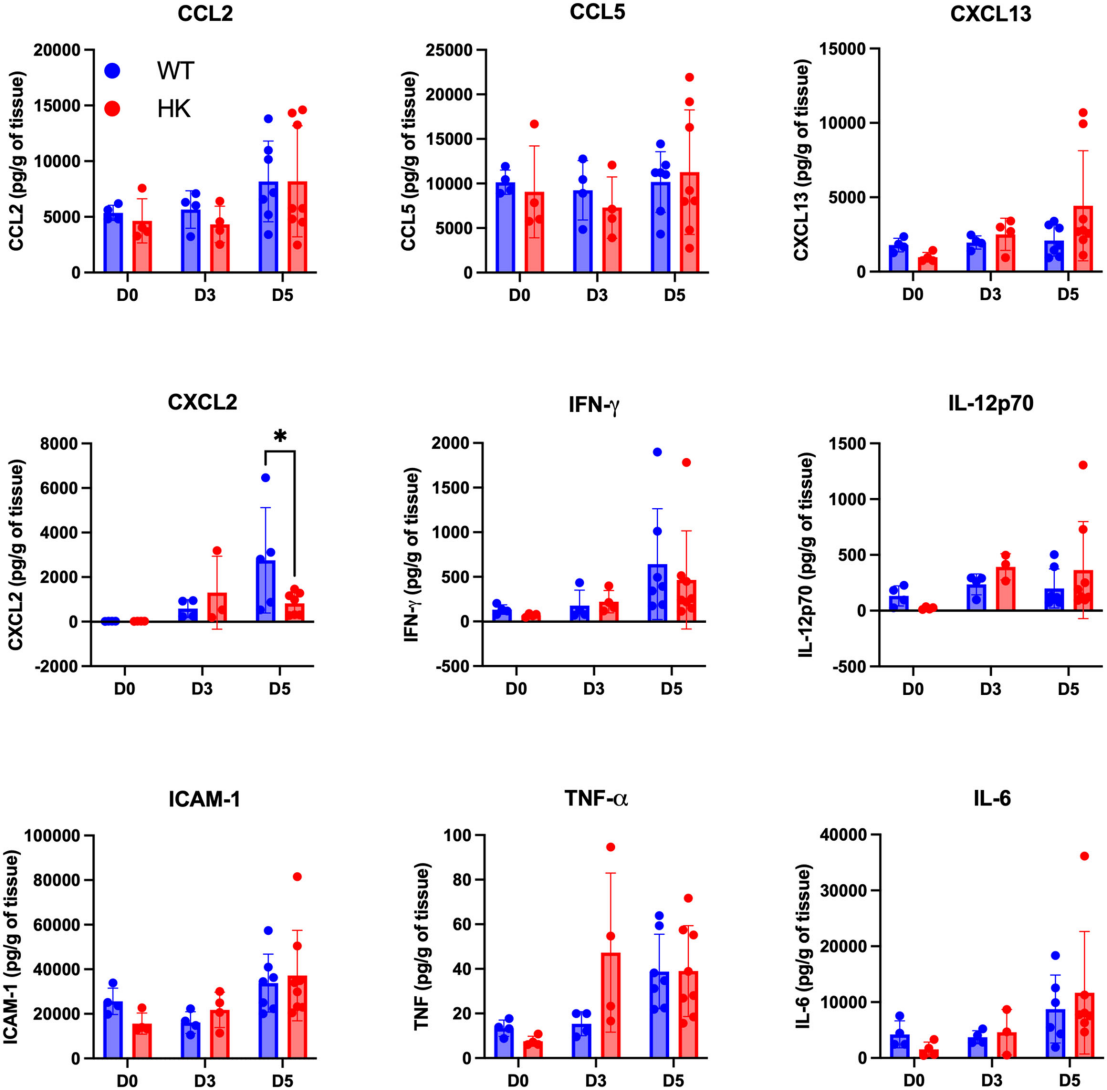
Mast cells modulate the chemokine milieu of the female reproductive tract following *Chlamydia* infection. Female reproductive tract homogenates from mast cell-deficient HK mice and littermate controls at baseline, D3 post-infection, and D5 post-infection were analyzed on a multiplex assay for soluble mediators, including chemokines and cytokines. Concentrations were normalized and graphed as per gram of tissue. Each dot represents an individual mouse. ^*^ *p* < 0.05, as determined by two-way ANOVA and Sidak’s multiple comparison test.

While the cytokine and chemokine milieu were mostly comparable between the mast cell-deficient and mast cell-sufficient mice, very interestingly, levels of CXCL2, a strong neutrophil chemoattractant, were significantly reduced in the mast cell-deficient mice (**Figure 6**), highlighting that mast cells modulate the chemokine milieu in an *in vivo* system as well.

### 3.7 Mast cells are important for the recruitment of neutrophils, eosinophils, and B cells at the early stages following *Chlamydia* infection

**Figure 7:**
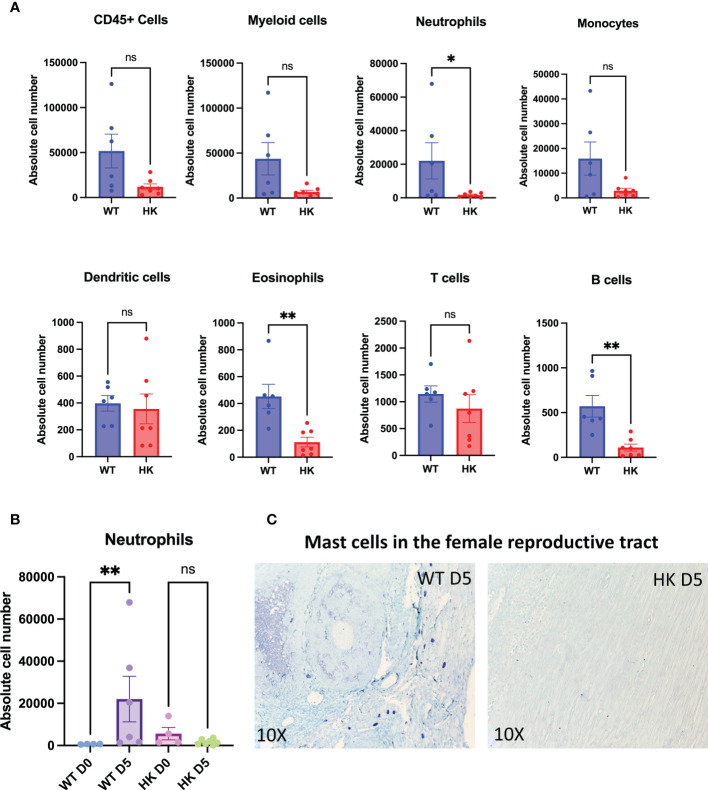
Mast cells are important for the recruitment of neutrophils, eosinophils, and B cells following *Chlamydia* infection. Uterine tract tissues following enzymatic digestion were processed into a single-cell suspension and analyzed for different immune cells by flow cytometry as described by the gating strategy shown in **Supplementary Figure 5**. Graphs show immune infiltration of CD45^+^ immune cells, myeloid cells, neutrophils, monocytes, dendritic cells, eosinophils, T cells, and B cells into the mouse uterine tracts **(A)**. *n* = 7 HKs and 6 littermate controls. ^*^ *p* < 0.05 and ^**^ *p* < 0.01, as determined by a Mann–Whitney *U* test. The neutrophil influx in the WTs and HKs at day 5 post-infection is shown in **(B)**. ^**^ *p* < 0.01, as determined by the Kruskal–Wallis test. The strategic location of mast cells in the mouse female reproductive tracts is visualized by toluidine blue staining **(C)**.

We hypothesized that the altered CXCL2 response could directly impair the recruitment of neutrophils into the uterine tract, potentially affecting the overall immune cell infiltrate. We, therefore, analyzed the infiltration of different immune cells into the uterine tract by flow cytometry with the gating strategy described in **Supplementary Figure S5**. While the total CD45^+^ immune cell population was comparable between the strains, the mast cell-deficient mice had significantly reduced numbers of neutrophils, eosinophils, and B cells compared to the mast cell-sufficient controls (**Figure 7A**). The numbers of infiltrating monocytes, dendritic cells, and T cells were comparable between the strains.

The influx of neutrophils was robust in the mast cell-sufficient controls at 5 days post-infection, and strikingly, mast cell-deficient mice completely lacked this response (**Figure 7B**). We also visualized intact mast cells in the myometrium and in close proximity to the oviducts (**Figure 7C**) at the acute time point post-infection, emphasizing their presence and influence on host responses to *Chlamydia*.

## 4 Discussion

**Figure 8:**
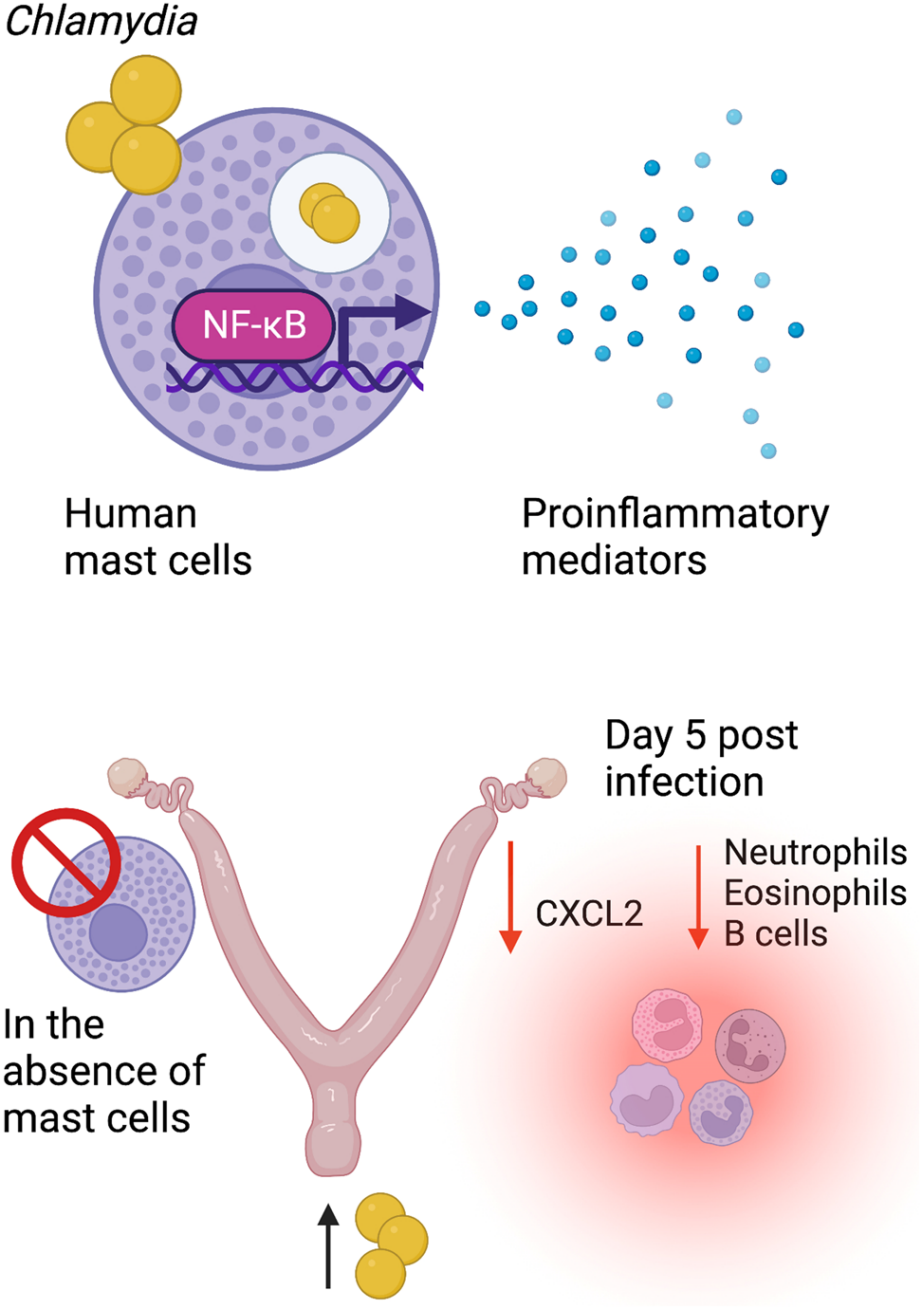
Mast cells selectively produce inflammatory mediators and impact the early responses to *Chlamydia* reproductive tract infection. Human mast cells sparsely take up *Chlamydia*, do not undergo degranulation, and are resistant to its intracellular life cycle. Mast cell–*Chlamydia* interaction occurs downstream of the NF-kB-mediated pathway with an upregulation of the expression of several inflammatory genes along with the production of proinflammatory mediators at the protein level. In the absence of mast cells *in vivo*, mice display an altered chemokine environment with reduced recruitment of effector cells, including neutrophils, eosinophils, and B cells into the reproductive tract.

This study suggests that human mast cells sparsely took up *C. trachomatis*, did not undergo degranulation in response to *C. trachomatis* exposure, and were not permissive to bacterial growth. However, in response to *C. trachomatis*, they became activated, exhibiting homotypic aggregation, expressing inflammatory genes *IL6*, *IL1B*, *CXCL8*, *CCL3*, and *NFkB1*, and producing several soluble mediators, including TNF, IL-1β, IL-1RA, IL-6, IL-23, CCL3, GM-CSF, CCL5, and CXCL8, which are all key players in acute inflammatory responses. These findings are in line with mast cells playing a sentinel role in *Chlamydia* infection, as has been described and reviewed for other bacterial infections (45). Mast cell–*C. trachomatis* interactions occurred at both mast cell surfaces and intracellular locations, potentially through a NF-κB-mediated pathway. IL-6 production occurred partially downstream of TLR2 signaling. These findings reflect a complex mast cell response to *C. trachomatis* that involves several host defense pathways. *In vivo*, mast cell-deficient mice displayed a dampened CXCL2 chemokine response in the uterine tracts following reproductive tract *Chlamydia* infection as well as reduced recruitment of neutrophils, eosinophils, and B cells during the peak of acute inflammation at day 5 post-infection. Taken together, these data indicate that mast cell functions are vital in shaping early immune and inflammatory responses to *Chlamydia* infection (see **Figure 8**).

*Chlamydial* DNA was found to be associated with mast cells at 3 h post-exposure to *C. trachomatis*. However, there was no increase in bacterial content between 24 and 48 h, the typical timeline required for a complete *chlamydial* life cycle in other cell types. To exclude the possibility that bacteria were only attached to the mast cell surface rather than being taken up by endocytosis, we assessed the location of *C. trachomatis* by electron microscopy. While control (McCoy) cells harbored substantial numbers of inclusion forming units (IFUs) at 6 and 40 h post-exposure to *C. trachomatis*, there were relatively very few IFUs in mast cells, even though they were exposed to a much higher infectious dose. This implied that the mast cells endocytosed *C. trachomatis* but were resistant to its subsequent replication. The high local level of mast cell proteases and other granule-associated mediators at the mast cell surface may also have impacted *C. trachomatis* uptake and subsequent bacterial growth.

*Chlamydia* spp. is internalized by a variety of host cells through a plethora of host–pathogen interactions. These include both low-affinity interactions with heparan sulfate proteoglycans and high-affinity bacterial ligand and host receptor interactions. Some of the established interactions include LPS binding to cystic fibrosis transmembrane conductance regulator, major outer membrane protein binding to mannose receptor, CT017 binding to β1 integrin, and polymorphic membrane proteins binding with epidermal growth factor receptor (2). Importantly, host immune activation in response to *Chlamydia* primarily occurs through exogenous and cytosolic sensing of chlamydial products by pattern recognition receptors including Toll-like receptors (46), NOD-like receptors (47), RIG-I-like receptors (48), or through inflammasome activation (49). Other innate immune signaling receptors such as the FPRs also play key roles in mucosal immune responses (26). TLR2 has been associated with peptidoglycans, macrophage inhibitory protein, and plasmid-regulated ligands, and specifically, TLR2 has been shown to interact with *chlamydial* major outer membrane protein as well as be associated with intracellular *chlamydial* inclusion bodies (50, 51). TLR4 has been established to bind with LPS and HSP60 and trigger downstream responses. On the other hand, *Chlamydia* has also evolved to have immune evasion mechanisms that specifically target the activation of these receptors (2, 52).

We chose to study the impact of FPRs and TLR2 based on our preliminary and broad survey of genes regulated in *C. trachomatis*-treated mast cells, recognizing that they were not likely to be exclusive pathways for mediator induction. In line with this model, most soluble mediators did not show a high degree of dependency on the function of these receptors. Of potential interest, the IL-6 response was substantially dependent on TLR2 activation. Intercepting TLR2 signaling selectively reduced the release of IL-6 protein but did not significantly alter IL-6 gene expression or the production of other inflammatory mediators. In a previously studied mouse model of *Chlamydia* infection, the complete lack of an IL-6 response resulted in less-severe tissue damage (53), but given the many roles of IL-6 in regulating immunity, the interpretation of the role of IL-6 at sites of acute infection remains undetermined. While the immune mechanisms leading up to reproductive tissue damage in *Chlamydia* infections remain unclear, our findings highlight the involvement of mast cells in soluble mediator production, including IL-6, and suggest that TLR2 modulation may provide a mechanism to modify this cytokine response.

Unlike dendritic cells, which in addition to endocytosis and antigen processing also get infected by *Chlamydia* (54), mast cells sense and respond to *Chlamydia* without substantial ongoing bacterial replication or degranulation. In other bacterial systems, such as *Salmonella typhimurium* and nonpathogenic *Escherichia coli*, mast cell degranulation has been shown to be inhibited by bacterial products (14, 55). In these models, when mast cell degranulation was permitted, an enhanced host defense response was observed. It is not known if *C. trachomatis* plays an active role in reducing mast cell degranulation. Notably, the *chlamydial* inclusion protein has been shown to intercept host cell machinery and interact with SNARE proteins (56). SNARE proteins are essential for mast cell secretion of preformed mediators, and potential immune evasion interactions could potentially contribute to the lack of mast cell degranulation.

Mast cells are known to be important for the recruitment of neutrophils to sites of infection (18) and for inducing protective immune responses against pathogens, including *Salmonella* sp., herpes simplex virus, and influenza virus (57, 58). Mast cell-derived IL-6 has been implicated in the promotion of survival in *Klebsiella* infection (59). In addition to producing inflammatory mediators and aiding in effector cell recruitment and the phagocytic activity of neutrophils, mast cells have been shown to produce extracellular traps and promote host defense against *Listeria* infection (27, 60, 61). Conversely, mast cells have also been reported to have detrimental roles. For example, increased mast cell counts and degranulation have been associated with severe SARS-CoV-2 virus infection (62). Thus, a lack of degranulation could potentially dampen mucosal immune responses to *Chlamydia* infection in a physiological setting but might also confer protection against chronic tissue scarring.

In keeping with a potential host defense role of mast cells in response to *C. trachomatis* infection, the majority of mediators induced in mast cells in response to *C. trachomatis* were chemokines or cytokines with roles in enhancing effector cell recruitment, particularly monocytes and neutrophils. Consistent with diverse clinical manifestations and disease outcomes of *Chlamydia* infection in women, we observed high donor-to-donor variability in certain mediator responses, such as the S100 proteins. This could potentially be attributed to genetic differences, although such analyses were beyond the scope of this study. The expression of several genes was downregulated following infection, including the chemokine receptor CXCR3 as well as pattern recognition receptors FPR2 and TLR4. The mechanism for these changes remains unclear.

The role of mast cells *in vivo* was also examined. Mast cells are distributed in the endometrium and myometrium of the human uterine wall, typically located toward the outer wall of the uterus in close proximity to the peritoneal cavity in mice (63). Following intravaginal inoculation, *Chlamydia* primarily infects and colonizes the murine mucosal epithelial cells. Epithelial cells and sentinel immune cells respond to pathogens by producing chemokines and cytokines, which results in the recruitment of early effector cells such as neutrophils and monocytes at the early stages of infection. Antigen-specific T cells infiltrate the reproductive tract in the later stages (4). T-cell-dependent interferon-γ responses are thought to be critical for controlling bacterial replication and eliminating infection (64, 65). Mice clear bacteria at around 4 weeks post-infection, but tubal damage still occurs as a result of chlamydial infection (66). Selective knockdown of immune signaling receptors, cytokines, and chemokines can modify this response (67).

The contribution of mast cells to the early immune responses to *Chlamydia* was investigated *in vivo* using the HK mast cell-deficient model. Mast cell reconstitution is often used to confirm mast cell dependency of responses (68). However, this is not possible at all mucosal sites (69). Adoptive transfer of cultured bone marrow-derived mast cells failed to effectively repopulate the female reproductive tract (data not shown). HK mice are a well-established *Kit* gene-independent model having specific deletions in the mast cell compartment and few recognized off-target effects. At baseline, in estrous cycle-synchronized mice, comparable numbers of CD45^+^ cells and subsets were observed in the female reproductive tracts of mast cell-sufficient and mast cell-deficient mice, implying that mast cell deficiency does not have a major impact on cellular infiltration in the absence of infection.

Following *Chlamydia* infection, mast cell-deficient mice displayed attenuated CXCL2 production, with the levels of other proinflammatory mediators including CCL2, CCL5, CXCL13, and effector mediators assessed as not being significantly altered. Human mast cells produced multiple soluble mediators *in vitro*. However, *in vivo*, multiple cells will contribute to the cytokine milieu. The low frequency of mast cells in the uterine tracts, their predominant location in the myometrium rather than the endometrium, contact times between mast cells and the bacteria, and the predominant role of epithelial cells all play into the outcomes of overall mediator production and host responses. The mast cell dependency of the CXCL2 response may suggest that mast cells are an unusually important source of this key chemokine for neutrophil recruitment.

In line with the diminished CXCL2 response, at day 5 post-*Chlamydia* infection, typically when an immune inductive site is developed at the submucosa, we observed a 34-fold increase in the neutrophils and a fivefold increase in the monocytes in wild-type controls, compared to very minimal changes in HK mice, highlighting a significant role for mast cells in the early recruitment of myeloid effector cells. Neutrophils have both protective and pathological roles in the outcomes of a *Chlamydia* infection. On the one hand, they can reduce bacterial impacts through phagocytosis, the production of reactive oxygen and nitrogen species, and the release of neutrophil extracellular traps. In contrast, the neutrophil release of MMPs and elastases has been positively correlated with chronic tissue damage (70). Eosinophil and B-cell numbers were also significantly lower in infected HK mice compared to wild-type controls. Eosinophils are reported to enhance stromal cell proliferation and prevent endometrial damage following *Chlamydia* infection (71).

Taken together, the reported human *in vitro* and mouse *in vivo* findings support the concept that mast cell functions are important in orchestrating early effector cell responses to *Chlamydia* infection. A key role for mast cell-mediated CXCL2 production is suggested. TLR2 appears to have a selective role in mediating IL-6 responses to *Chlamydia*. Through a better understanding of these responses and the subsequent events that lead to tissue damage, we can begin to design improved approaches to combat the negative impacts of *Chlamydia* infection.

## Data availability statement

The raw data supporting the conclusions of this article will be made available by the authors, without undue reservation.

## Ethics statement

The studies involving human participants were reviewed and approved by IWK Health Centre Research Ethics Board. The patients/participants provided their written informed consent to participate in this study. The animal study was reviewed and approved by Dalhousie University, University Committee on Laboratory Animals.

## Author contributions

AM, ES, JW and JM conceived of the study. AM and ES conducted the experiments and analyzed and interpreted data. IH provided intellectual support for CBMC experiments and flow cytometry data analysis. JW provided methodological design support for infection studies and JM provided methodological design support for mast cell studies. AM and JM prepared the manuscript. JW and JM were co-supervisors of graduate students ES and AM. All authors edited and approved the final version of this paper to be published and agree to be accountable for all aspects of the work in ensuring that questions related to the accuracy or integrity of any part of the work are appropriately investigated and resolved. All authors contributed to the article and approved the submitted version.
