# Supplementary material for: Mast cells selectively produce inflammatory mediators and impact the early response to Chlamydia reproductive tract infection
Source: Front Immunol. 2023 Apr 17;14:1166068. doi: 10.3389/fimmu.2023.1166068 (PMC10150091; doi:10.3389/fimmu.2023.1166068)
Supplement: Supplementary file 1 [file Table_1.docx]

**Supplementary Table 1. List of antibodies used in flow cytometry studies**

| **Marker** | **Specificity** | **Fluorophore** | **Clone** |
| --- | --- | --- | --- |
| CD117 | Human | APC | 104D2 |
| CD117 | Human | PE | 104D2 |
| TLR2 | Human | PE | TL2.1 |
| TLR4 | Human | PE | HTA125 |
| FPR2 | Human | PE | REA663 |
| CD11b | Human | FITC | ICRF44 |
| CD11a (LFA-1α) | Human | FITC | HI111 |
| CD54 (ICAM-1) | Human | PE | HCD54 |
| CD49b | Human | APC | P1E6-C5 |
| CD49c | Human | PE | ASC-1 |
| CD117 | Mouse | PE | 2B8 |
| CD45R/B220 | Mouse | BV786 | RA3-6B2 |
| Ly-6G | Mouse | BV480 | 1A8 |
| CD11c | Mouse | BB700 | HL3 |
| Ly-6C | Mouse | PE-Cy7 | HK1.4 |
| Siglec-F | Mouse | PE-CF594 | E50-2440 |
| CD8a | Mouse | APC-H7 | 53-6.7 |
| CD19 | Mouse | R718 | 1D3 |
| F4/80 | Mouse | BUV805 | T45-2342 |
| CD11b | Mouse | BUV563 | M1/70 |
| I-A/I-E (MHCII) | Mouse | BUV395 | 2G9 |
| CD3e | Mouse | BUV661 | 145-2C11 |
| CD45 | Mouse | BUV496 | 30-F11 |
| Fixable viability dye |  | FVS575V |  |
