# Supplementary material for: Mast cells selectively produce inflammatory mediators and impact the early response to Chlamydia reproductive tract infection
Source: Front Immunol. 2023 Apr 17;14:1166068. doi: 10.3389/fimmu.2023.1166068 (PMC10150091; doi:10.3389/fimmu.2023.1166068)
Supplement: Supplementary file 2 [file Table_2.docx]

**Supplementary Table 2. List of genes screened in the PCR Array**

| Chemokine receptors | *CCR1, CCR2, CCR3, CCR5, CCR6, CCR7, CCR8, CXCR1, CXCR2, CXCR3, CXCR4, CXCR6, CX3CR1, CXCL10* |
| --- | --- |
| Cytokines and cytokine receptors | *IL1A, IL1B, IL1RN, IL2, IL4, IL5, IL6, IL6R, IL6ST, IL10, IL13, IL17A, IL18, IL22, IFNG, TNF, TNRSF1A, TGFB1* |
| Chemokines | *CCL2, CCL3, CXCL8, CXCL2* |
| Complement pathway | *CRP, C2AR1, C5AR1* |
| Adhesion molecules | *ICAM1, VCAM1, ITGB2, ITGAM* |
| Pattern recognition receptors | *FPR2, TLR2, TLR4, CD14, MBL2, CD163* |
| Signalling molecules | *MAPK1, MAPK14, AKT1, NFKB1, STAT3, RELA, GATA3* |
| Acute phase proteins | *A2M, F2, F2R, F3, TF, HBA1, HBA2* |
| Metabolic molecules | *PTGES, INS, MO, ELANE, LBP, FCGR1A, APOE, APOA1, CALCA, S100A8, S100A9, SERPINA1, SERPINA3, AHSG, CEBPB, BDKRB1, DEFB1, HP, LP, SAA1, F2R1* |
| Reference genes | *TBP, GAPDH and HPRT1* |
