# Supplementary figures and images for: Mast cells selectively produce inflammatory mediators and impact the early response to Chlamydia reproductive tract infection
Source: Front Immunol. 2023 Apr 17;14:1166068. doi: 10.3389/fimmu.2023.1166068 (PMC10150091; doi:10.3389/fimmu.2023.1166068)

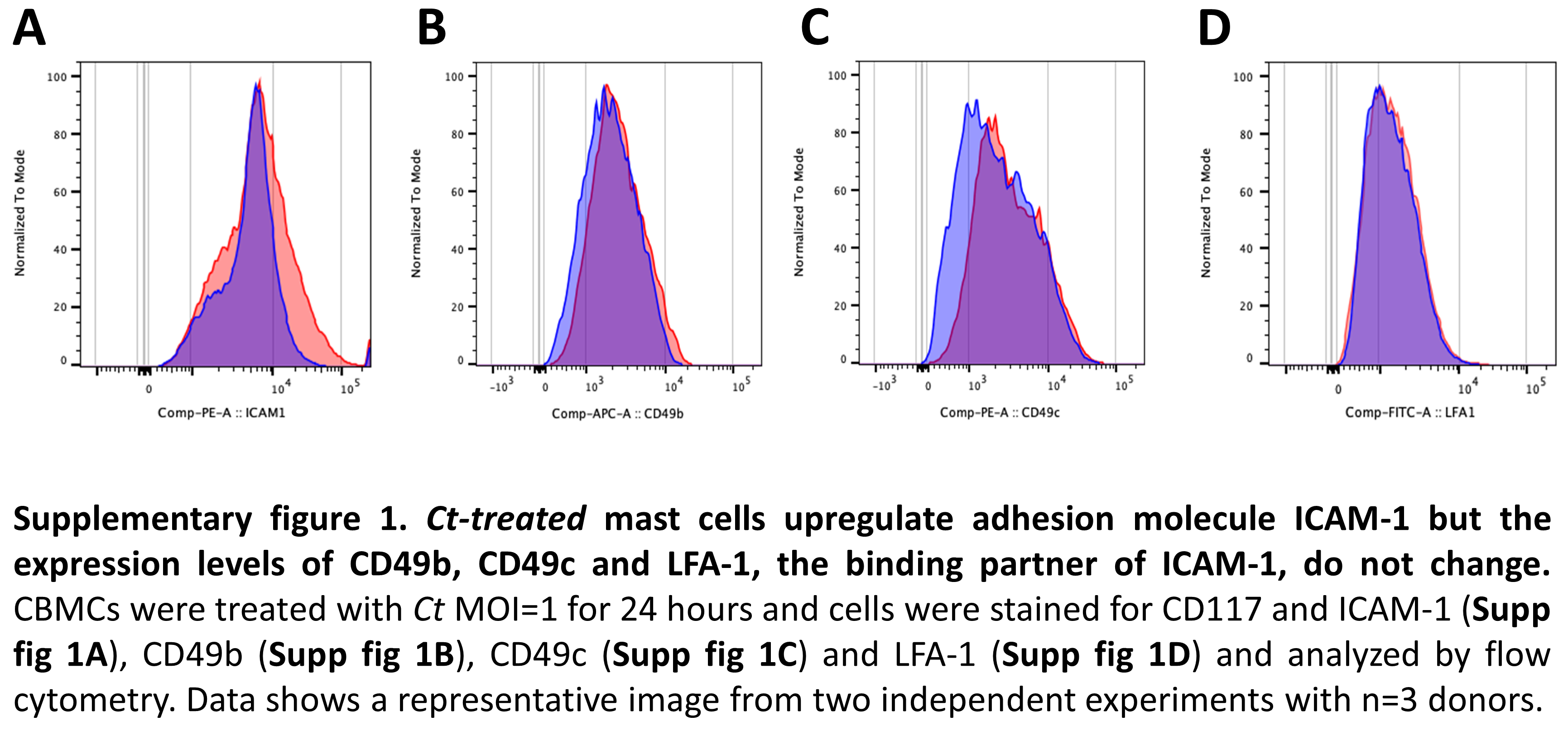

Supplement: Supplementary file 3 [file Image_1.tif]

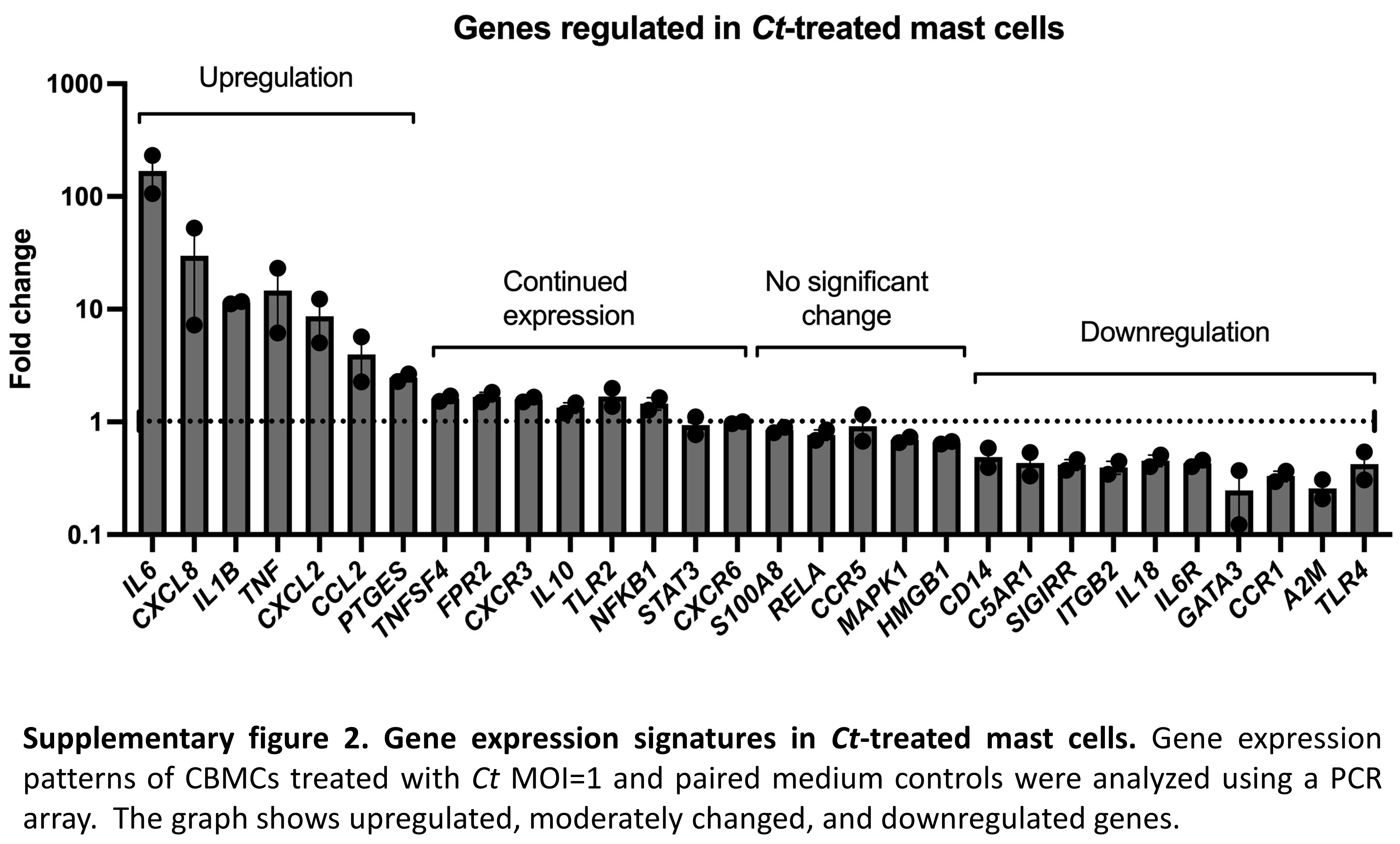

Supplement: Supplementary file 4 [file Image_2.tif]

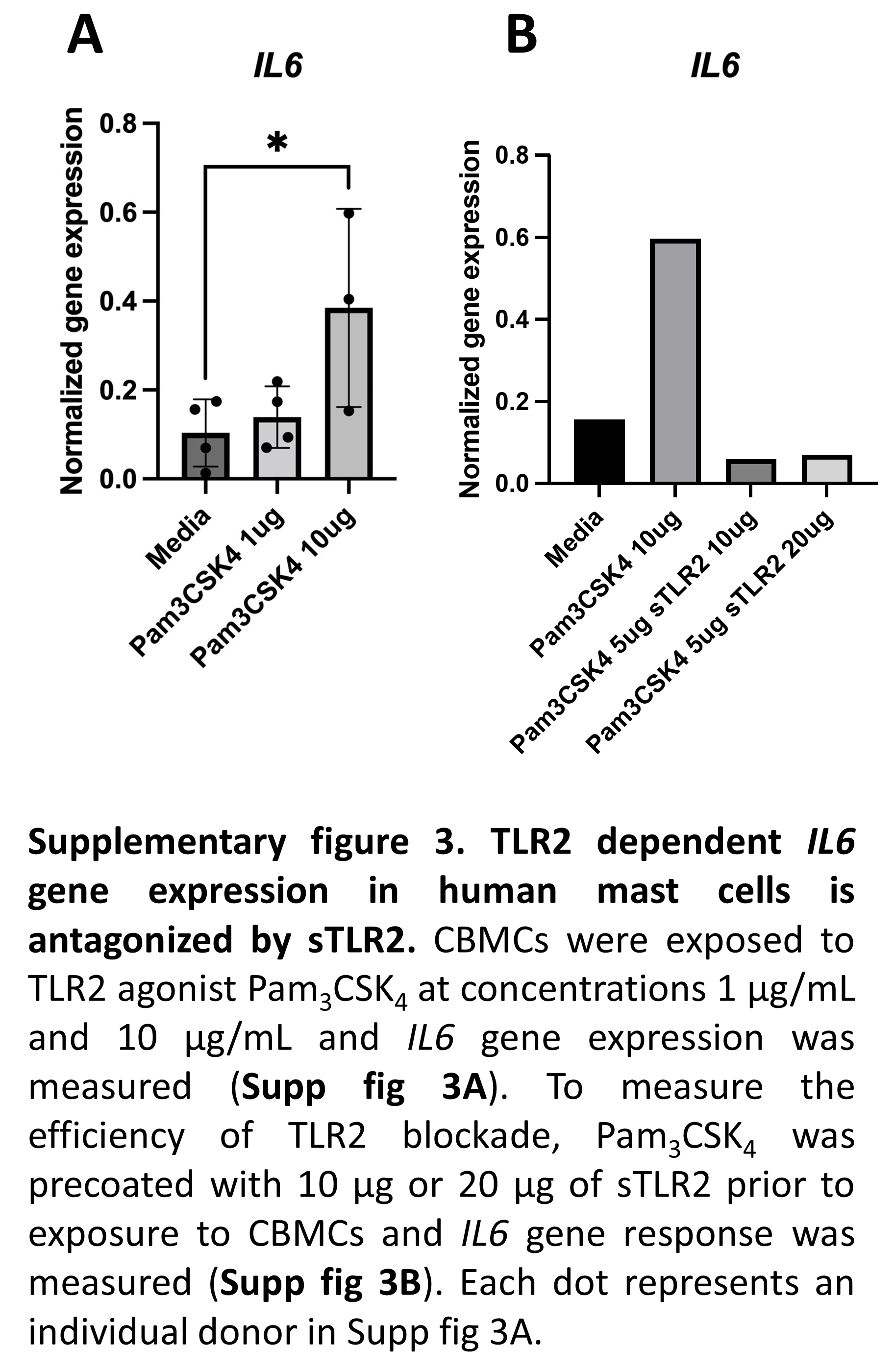

Supplement: Supplementary file 5 [file Image_3.tif]

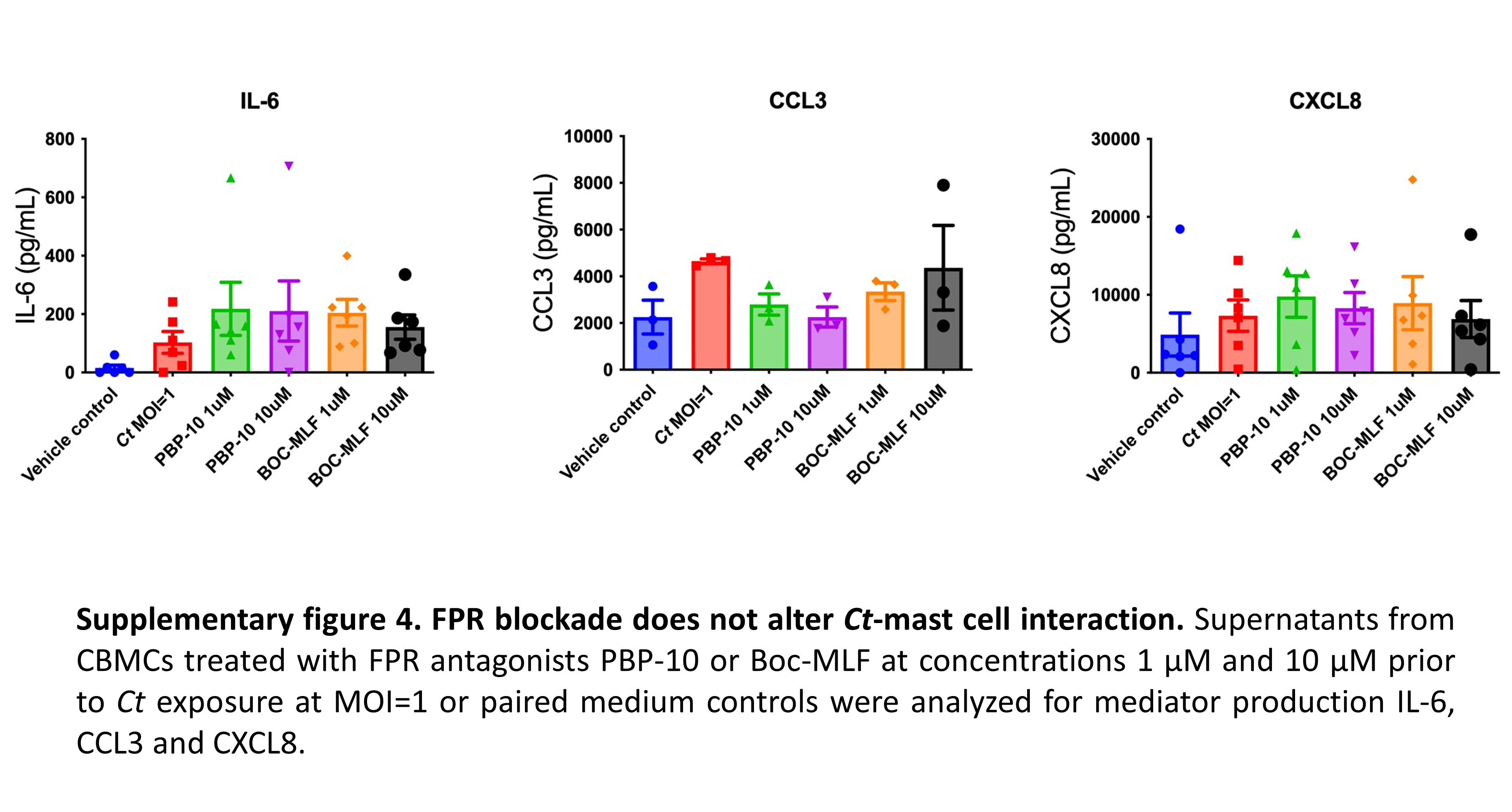

Supplement: Supplementary file 6 [file Image_4.tif]

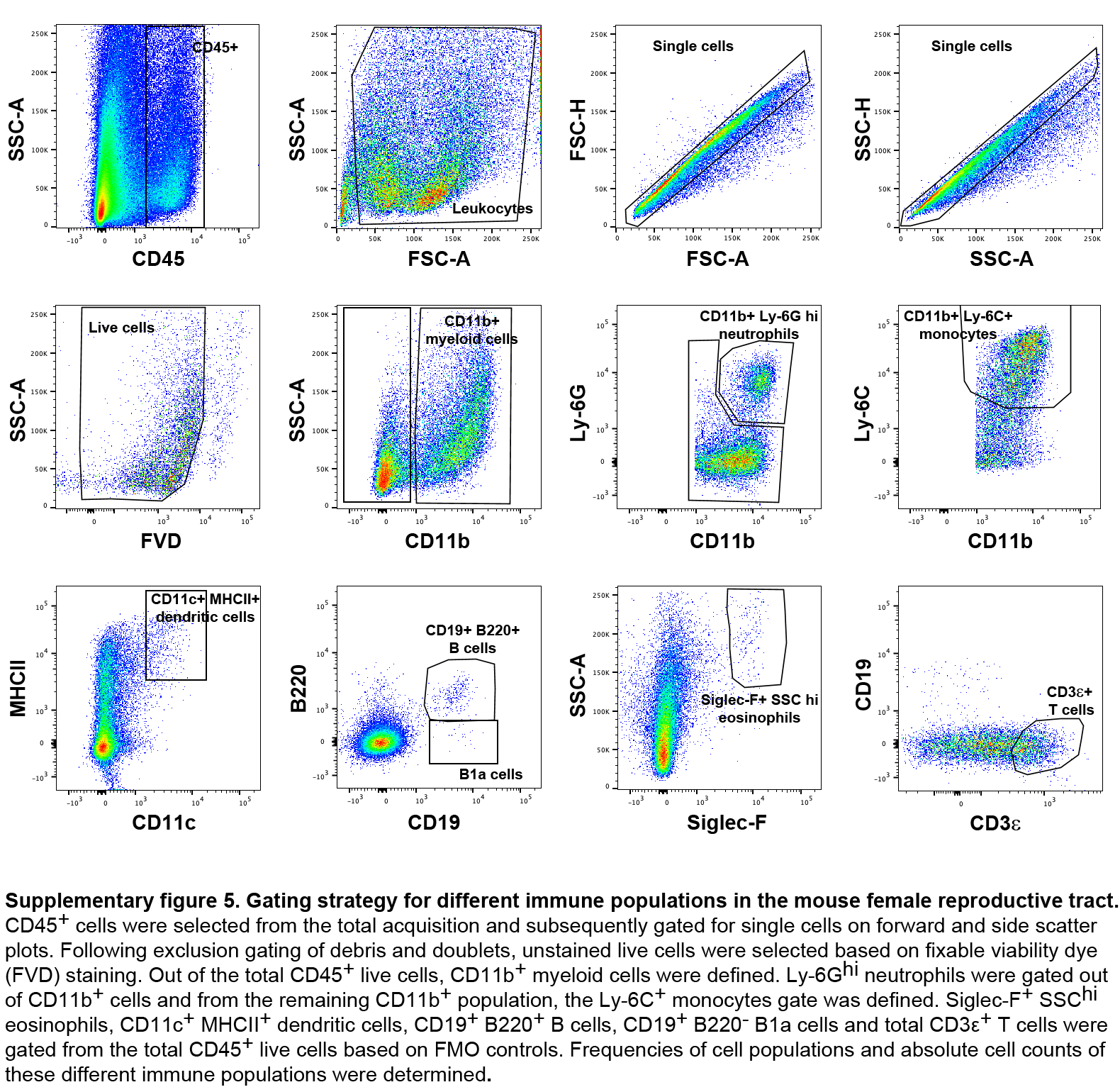

Supplement: Supplementary file 7 [file Image_5.tif]
